# Among Those You Know

**Published:** 1897-08

**Authors:** 


Secretary.

### AMONG THOSE YOU KNOW.

Lawson N. Fuller, seventy-five years old, drove eight horses a full mile over Fleetwood Track in 3.18¼; six-in-hand in 2.58. These horses were all bred, raised, and trained by Mr. Fuller.

Dr. Jackson has given up practice in New York City and taken a position to manage a stock-farm near Albany.

Charles F. Spies, V.S., has located at Bayonne, N. J.

T. E. Smith, V.S., has located at No. 309 Barrow Street, Jersey City, N. J.

Dr. Abel, of the Bureau of Animal Industry, has been transferred from Buffalo to Brooklyn; Dr. Murphy from Brooklyn to New York (east-side abattoirs); Dr. L. P. Cook from east-side abattoirs to west-side; Dr. E. L. Volgenau from New York City to Buffalo.

Dr. Huidekoper and his wife are at Newport. Mrs. Huidekoper is doing well.

Dr. D. W. Cochran has been on a visit to Toronto, calling at Prof. Smith's, also at Drs. Hinkley and Claris's at Buffalo on his way returning.

Dr. Thomas Giffin has for some time been affected with threatened locomotor ataxia. On the recovery of his wife, the doctor and his family leave for the mountains.

Drs. Heard, O'Shea, and J. S. Cattanach will spend the summer at Newport; Drs. C. C. Cattanach and Dickson at Long Branch.

Dr. A. J. Sheldon, of Boston, Mass., was one of the applicants before the civil service commission in April last.

Dr. H. B. Adair is now located at Nogales, Arizona, as assistant inspector of the Bureau of Animal Industry along the Texas-fever quarantine line.

Dr. W. C. Rayen, of Nashville, Tenn., of the quarantine department of the Bureau of Animal Industry, has been changed to the meat-inspection department, and directed to report at Chicago, September 15th.

Dr. Fred. de M. Bertram, former house-surgeon of the Veterinary Department of the University of Pennsylvania, has gone to Newport, R. I., for a summer location in practice.

Veterinarian J. F. Deadman's wife, of Sault Ste. Marie, Mich., gave birth to three healthy boys early in July.

Dr. Charles A. Schauffler fills the *rôle* of veterinarian to the Woman's Branch of the Society for the Prevention of Cruelty to Animals in the up-town district of Philadelphia.

Dr. Wm. G. Shaw, house-surgeon Veterinary Department of the University of Pennsylvania, was a visitor to Pittsburg early in July.

Dr. Alex. Glass, of Philadelphia, fills the *rôle* of official photographer of the Corinthian Yacht Club, of which he is an ardent devotee, and with true nautical devotion pilots his many friends into all the creeks and coves of the beautiful Delaware River in his naphtha launch, "Corona."

**See if the railroad does not favor you.**

Dr. W. W. Phipps, of Lionville, Chester Co., Pa., has been selected a starting judge of the Downingtown Driving Association.

Dr. F. Rawson, of Lawton, Mich., takes much interest in the breeding of collie dogs.

Dr. Leonard Pearson was a visitor to Boston in July, and spent some hours at the Deer Foot Farms.

Dr. C. E. Stewart, of Chariton, Iowa, holds the position of Assistant State Veterinarian.

Dr. John C. Stewart, of Danville, Ill., fills the *rôle* of Assistant State Veterinarian for his district.

**Have you sent in an application for membership?**

Dr. Walter L. Hart, of Philadelphia, has received the appointment of City Veterinarian. He for many years assisted his brother, the late Dr. John R. Hart. Dr. Walter L. Hart is a graduate of the American Veterinary College. He is well equipped for the position, and will no doubt fill it with increasing benefit to the profession and credit to himself.

Dr. A. N. Lushington, of Philadelphia, successfully passed the examination of the Virginia State Board of Veterinary Medical Examiners.

Dr. E. F. McLean, of Cincinnati, the noted turfman, died suddenly on July 29th, from heart-disease, at the Oakley running track.

Dr. M. R. Trumbower, of Sterling, Ill., has gone to Colorado temporarily, hoping thereby to benefit his wife's health.

Dr. John W. Connoway, of Columbia, Mo., was a recent visitor to Kansas City, securing material while there for the further study of Texas-fever.

Dr. J. C. Norton, Territorial Veterinarian for Arizona, recently visited the national capital and many of the large cattle-markets in the interests of the Arizona Cattle Commission.

Dr. Charles T. Goentner, of Bryn Mawr, Pa., is seriously ill from the combined injury by a horse-kick in the jaw, causing a tetanic condition of the latter, and a prior condition of threatened appendicitis, for which he was being treated when he received the above injury.

Dr. J. A. Pearson, of Philadelphia, has opened an infirmary in conjunction with his private practice.

Dr. W. J. Hinman represented the Manitoba Horse Breeders' Association at the Winnipeg Industrial Exhibition Association in July.

James E. Duncan, V.S., has taken a medical course, and is now practising human medicine and surgery in Pittsburg, Pa.

Dr. William R. Howe, of Dayton, Ohio, is working hard for the establishment of meat- and milk-inspection in that city.

Dr. M. J. Henderson, of Syracuse, N. Y., was one of the judges on the stand at the recent races at Kirk Park, where the stallion Bel Bel died from exhaustion after being denied the privilege of being withdrawn.

Dr. R. S. Huidekoper, of Newport, R. I., has been selected by the fashionable patrons of that famous resort as the promoter of a dog-show to be held in August. It will be held on a site near the Casino.

Veterinarian H. C. Moore, of Albany, N. Y., is summering at the "Plaza" at Asbury Park, N. J.

Dr. R. S. Huidekoper is Secretary-Treasurer of the Bull Terrier Club of America.

Drs. Huidekoper, Pearson, Trumbower, Sherwood, Peters, and Roberts have been appointed by the American Kennel Club as an advisory committee to procure statistics and report on the subject of rabies in America.

Thomas J. Cooper, V.S., who recently located at Paterson, N. J., has been seriously ill, but is now on the way to recovery.

